# Increased rise time of electron temperature during adiabatic plasmon focusing

**DOI:** 10.1038/s41467-017-01802-y

**Published:** 2017-11-21

**Authors:** Olga Lozan, Ravishankar Sundararaman, Buntha Ea-Kim, Jean-Michel Rampnoux, Prineha Narang, Stefan Dilhaire, Philippe Lalanne

**Affiliations:** 1 Laboratoire Onde et Matière d’Aquitaine (LOMA), UMR 5798, CNRS-Université de Bordeaux, 33400 Talence, France; 2 Department of Materials Science and Engineering, Rensselaer Polytechnic Institute, Troy, NY 12180 USA; 3 Laboratoire Charles Fabry (LCF), UMR 5298, CNRS-IOGS-Université Paris XI, Institut d’Optique, 91120 Palaiseau, France; 4 Faculty of Arts and Sciences, Harvard University, Cambridge, MA 02138 USA; 5 Laboratoire Photonique, Numérique et Nanosciences (LP2N), UMR 5298, CNRS-IOGS-Université de Bordeaux, Institut d’Optique d’Aquitaine, 33400 Talence, France

## Abstract

Decay of plasmons to hot carriers has recently attracted considerable interest for fundamental studies and applications in quantum plasmonics. Although plasmon-assisted hot carriers in metals have already enabled remarkable physical and chemical phenomena, much remains to be understood to engineer devices. Here, we present an analysis of the spatio-temporal dynamics of hot electrons in an emblematic plasmonic device, the adiabatic nanofocusing surface-plasmon taper. With femtosecond-resolution measurements, we confirm the extraordinary capability of plasmonic tapers to generate hot carriers by slowing down plasmons at the taper apex. The measurements also evidence a substantial increase of the “lifetime” of the electron gas temperature at the apex. This interesting effect is interpreted as resulting from an intricate heat flow at the apex. The ability to harness the “lifetime” of hot-carrier gases with nanoscale circuits may provide a multitude of applications, such as hot-spot management, nonequilibrium hot-carrier generation, sensing, and photovoltaics.

## Abstract (web summary)

Knowledge of the electron-gas dynamics in nanometric hot spots is of importance for hot-carrier technologies. Here Lozan et al. present a theoretical and experimental analysis of the spatio-temporal dynamics of hot electrons in a nano-focusing surface-plasmon polariton taper.

## Introduction

Light incident on metallic nanostructures excites surface-plasmon polaritons (SPPs), which may decay to hot carriers through several mechanisms including direct interband transitions, phonon-assisted intraband transitions, and geometry-assisted transitions^2,3^. This rapid decay of SPPs to electron–hole pairs on femtosecond time scales is detrimental to most plasmonic applications and devices relying on huge local field enhancements, since it destroys the coherence of the collective electron oscillations. Conversely and positively, plasmonic hot carriers provide new opportunities for nanoelectronic and nanophotonic applications, such as energy conversion, spectroscopy, and sensing. In particular, hot carriers that are generated in the vicinity of the surfaces of plasmonic nanostructures and which reach their highest temperature in a few hundreds of femtoseconds can be extracted before their energy is lost. These carriers may be useful for a number of applications including enabling novel optoelectronic device functionality^1–4^, for localized heating in medical applications^5,6^, to boost photocatalytic activity for chemical synthesis and solar fuel generation^7–9^, for high-efficiency gapless photovoltaic energy conversion^1,3,4,10–13^, and to enable new high-resolution-imaging instruments^14^. Consequently, understanding the spatiotemporal dynamics of plasmonic hot carriers is highly desirable, particularly in devices capable of generating them with high-efficiency and nanoscale localization in “hot spots” where they may be extracted easily.

Despite recent significant progress, there are still many open questions that need to be addressed to enable reliable and widespread application of plasmon-generated hot carriers. A key challenge that remains is that the generated hot carriers thermalize rapidly and lose energy to thermal carriers and phonons in the material. The possibility of extending hot-carrier lifetimes and controlling the interfacial spatial and temporal distribution of hot carriers in nanoscale devices is critical, as this would provide opportunities to modify charge transfer processes at the nanoscale and to realize reactive nanostructures for efficient surface chemistry applications, for example.

So far, the relaxation dynamics of hot-carrier gases has been studied for simple geometries, mostly metallic nanoparticles or thin metallic films, and hot-carrier gas lifetimes of typically a few hundreds of fs have been recorded^15^. In contrast, here, we study the SPP-to-hot-electron conversion in a nanofocusing SPP taper geometry^16^. This geometry is a standard motif in plasmonic circuitry for its efficacy to focus electromagnetic fields to a single hot spot, but surprisingly, its performance for hot-carrier generation and extraction has not yet been analyzed, either experimentally or theoretically. Additionally, the nanofocusing SPP taper represents a facile test bed for unambiguously investigating the impact of confinement on the dynamics of the generated hot electrons, since it offers a nearly continuous transformation from an extended metal film to a nanoconfined geometry at the apex of the taper.

The knowledge of the electron gas dynamics in nanometric hot spots is of crucial importance for hot-carrier technologies, but it requires the deployment of high temporal and spatial resolution techniques that reveal absorption losses by probing the electron temperature at the relevant short time and length scales. We use the well-known time-domain thermoreflectance technique to record a temporal sequence of images of the local heat source, or equivalently of the hot-carrier density generated by plasmon decays, with 100-fs time and 400-nm spatial resolutions^17–20^.

Our thermoreflectance measurements find a strong (×30) enhancement of the absorption as the launched SPPs approach the tip apex, compared to the extended film. Unexpectedly, they also reveal a substantial increase of the lifetime of the hot electron gas temperature at the apex. Using theoretical models, here, we attribute this increase to two contrasted effects linked to confinement, a squeezing of the diffusion processes due to the initial spatial distribution of the generated hot carriers, and a backward heat flow from the apex toward cooler regions of the antenna. This interpretation provides basic design clues to create long-lived hot-electron baths, which are important for applications where hot electron generation and sustained high carrier temperatures are beneficial^1–4^.

## Results

### Sample design and fabrication

**Fig. 1:**
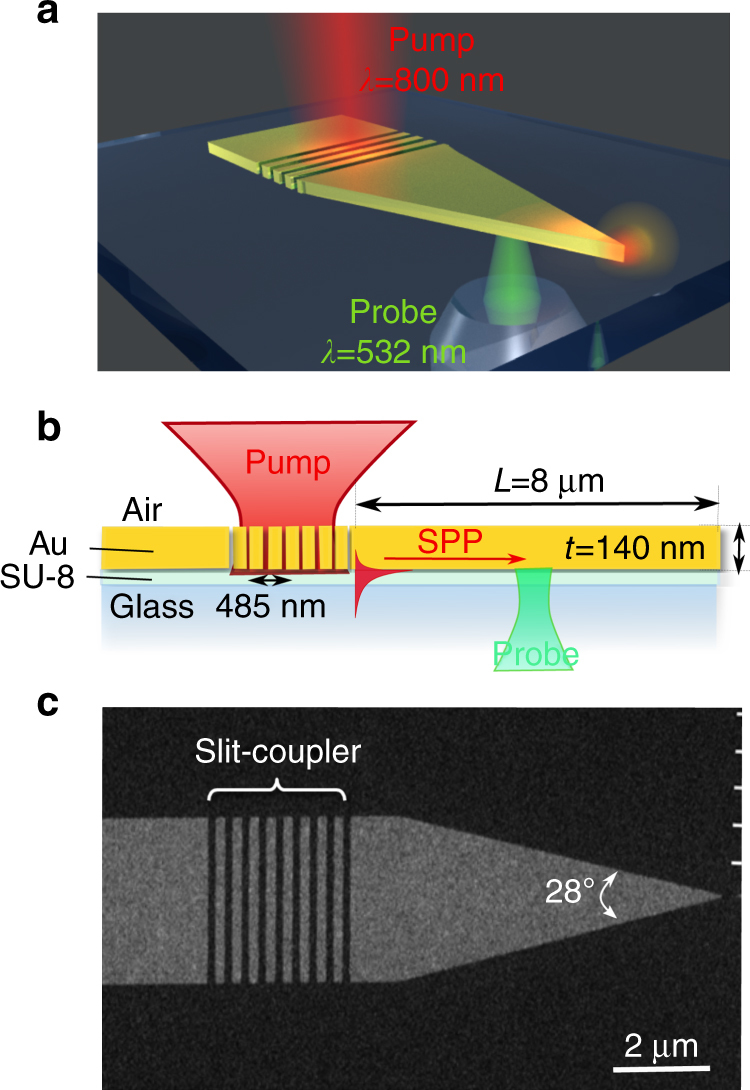
The plasmonic coupler–taper platform. **a** A pump pulse is focused from air onto a slit array, and launches SPPs at the dielectric/Au interface that propagate toward the tip apex. The SPP damping is probed by a delayed focused probe beam shown incident from the substrate. **b** Line shape of the sample. It shows how an SPP can be generated at the dielectric/Au interface and focused adiabatically toward the tip apex. **c** Scanning electron microscope top view of the device

The plasmonic coupler–taper device used in this study is composed of a laterally tapered metal stripe waveguide and a slit-array SPP-coupler optimized for operation with a Gaussian pump-beam (*λ*_0_ = 800 nm, beam-waist ≈1.6 µm) polarized perpendicularly to the slits (TM polarization), see Fig. 1. Since some measurements are performed with a high fluence (≈10 J/m^2^), the coupler is designed for a weak-absorption operation with a relatively high SPP-launching efficiency, to lower the risk of sample damages. For the optimization, we assume that the Gaussian pump-beam is illuminating the sample at normal incidence, either from air or from the substrate and that the SPP is launched at the dielectric/Au interface with a wavelength $\lambda _{{\mathrm{SP}}} \approx \lambda _{\mathrm{0}}{\mathrm{/}}1.5$, since the air/Au SPP is cutoff before reaching the tip apex^20^. We also consider regularly spaced arrays of identical slits for the design, the period *a* being fixed by the grating equation *a* = *λ*_SP_ = 485 nm.

The coupler design is performed by first computing the electromagnetic field scattered by the pump beam using the Au permittivity *ε*_Au_ = −26 + *i*1.84^22^, and then by rigorously determining the SPP-launching efficiency with an exact modal overlap integral^23^ computed with a near-field to far-field transformation freeware^24^. This procedure is then repeated iteratively, varying the number *N* of slits, the slit width *w*, and the Au-film thickness *t*, all independently. For the optimization, we use the simplex search Nelder–Mead method^25^. We found that much higher launching efficiencies are achieved when the pump-beam illuminates the coupler from air, and that a weak coupler-absorption of only 14% with a reasonably good SPP-launching efficiency of 30% is achieved for *N* = 8, *w* = 100 nm, and *t* = 140 nm.

We use a laterally tapered metal stripe waveguide with an apex angle of 28° and a length of *L* = 8 μm, a value comparable to the SPP propagation decay length (≈10 μm) at *λ*_0_ = 800 nm. Similar geometries have been recently characterized by detecting upconversion luminescence from erbium ions implanted in the substrate^21^. There are two ideas underlying the adiabatic nanoconcentration of the optical energy in the taper. The launched SPP is initially focused by the geometrical reduction of the metal stripe width. This focusing is analogous to that obtained by a lens with a classical optical beam and provides SPP confinements down to dimensions ≈*λ*_SP_ limited by diffraction. Then, as the SPP at the SU-8/Au interface enters the last 100 nm of the taper apex, it progressively slows down with a group velocity *v*_g_ proportional to the separation distance *x* from the apex, *v*_g_ ~ *ωx* ^15^. Since the electric field scales as $v_{\mathrm{g}}^{ - 1}$, a very strong absorption with a universal scaling ∝*x* ^−2^ close to the apex is expected. This critical aspect will be documented hereafter with electromagnetic computations.

The sample is made of a glass substrate coated with a polymerized 1-µm-thick SU-8 photoresist film used for better adhesion of gold. The 140-nm-thick Au film is deposited by Ar-magnetron plasma sputtering, and then coated with an electrosensitive resist layer used for writing the taper and coupler patterns with electron beam lithography. The resist pattern is transferred in the Au layer by argon-ion beam etching.

### SPP propagation and decay in the taper

**Fig. 2:**
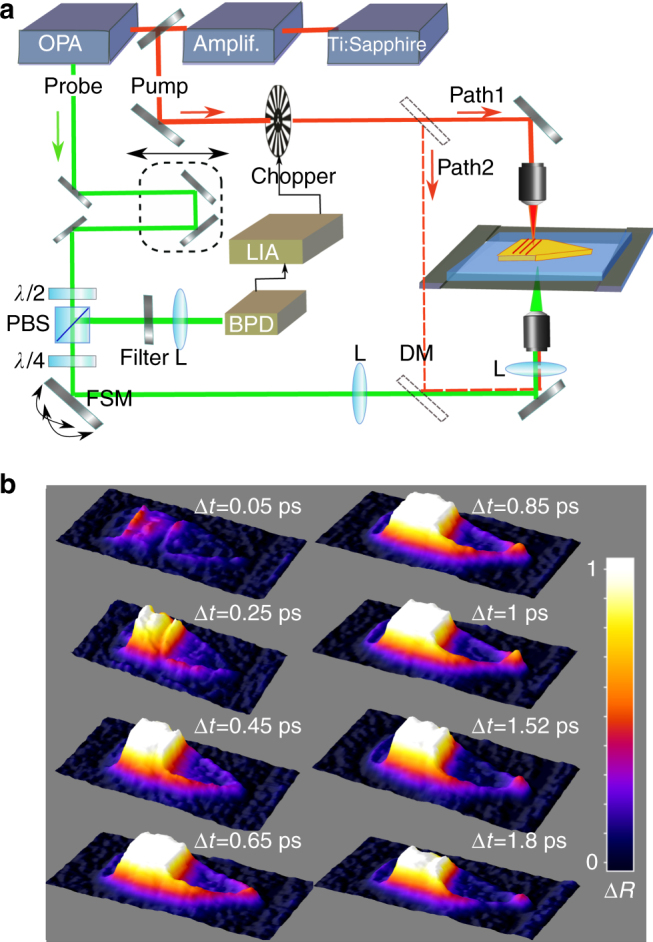
Thermoreflectance measurements. **a** Sketch of the time-domain thermoreflectance setup: PBS polarizing beam splitter, λ/4 and λ/2 quarter- and half-waveplates, BPD balanced photodiode, DM dichroic mirror, FSM fast-steering mirror, L lens, LIA lock-in amplifier. **b** Reflectance-variation (Δ*R*) images recorded at different pump–probe delay times, Δ*t* = 0.05, 0.25… and 1.8 ps. The image series shows the initial SPP launching followed by propagation along the taper, and eventually the formation and disappearance of a bright spot at the apex. The 16 × 6 μm^2^ saturated images are presented using the same color scale. A movie is available in Supplementary Movie 1

In the experiment sketched in Fig. 2a, the tiny variations Δ*R* of the reflectivity are related to tiny changes of the dielectric function that results from electron–hole pairs generated by the ultrafast decay of the launched SPPs and by the change of the occupancy of electronic states close to the Fermi level. The variations are recorded by a time-domain thermoreflectance setup based on two delayed pump- and probe-focused beams delivered by an amplified Ti-sapphire laser system seeding an optical parametric amplifier^18^. The pump pulse (150-fs FWHM, 250-kHz repetition rate, *λ*_0_ = 800 nm, 200-μW average power, 1 nJ per pulse, and 40 GW/cm^2^ peak intensity) is TM polarized. The circularly polarized probe pulse is delayed with two retroreflectors mounted on a translation stage. Its photon energy is chosen to be slightly lower than the transition energy from the *d*-band to the Fermi energy (*λ* = 532 nm, 30-μW average power), which guarantees a good sensitivity and a proportionality between Δ*R* and the temperature variations of the electron gas^26^. In the experiment, the probe beam can be focused on the taper from either the air cladding or the substrate. A fast-steering mirror allows to raster scan the sample and to record images with a ≈400-nm spatial resolution limited by the probe waist.

Figure 2b shows a set of raw Δ*R* images recorded at the SU-8/Au interface for eight values of the pump−probe delay, Δ*t* = 0.05, … 1.8 ps. The sign of Δ*R* is negative in our measurements, since smearing the state occupancy causes an increase of absorption at our probe wavelength^27^, but we conveniently plot the absolute value throughout the paper. A movie of the SPP launching and nanofocusing with a 67-fs time step is available in Supplementary Movie 1. First of all, we note that the typical time scales (~500 fs) for which the thermoreflectance dynamics is markedly impacted are much larger than the delay (~45 fs) related to the SPP transit time over the 8-µm taper length. Indeed, the dynamics of the hot-carrier generation and relaxation, including electron–electron, electron–phonon, and electron–surface interactions, is revealed by the thermoreflectance records.

Three other important observations can be made. First, large Δ*R* values are observed at the lateral edges of the taper. This absorption, which significantly varies with the focusing position of the pump beam on the coupler and is systematically observed in all our measurements, is due to additional decay channels for the SPPs at the etched edges. Second, a bright spot at the tip apex, which is particularly intense for the frame recorded at Δ*t* = 1 ps, is observed at the tip apex. Finally, inside the taper away from the edges, Δ*R* is much weaker than at the lateral edge and at the tip apex, suggesting that the SPP decay occurring during the propagation along the taper is not the dominant mechanism for the absorption.

Note that the present measurements strongly differ from direct steady-state temperature mapping of nanofocusing tapers recently reported with classical low temporal-resolution techniques^28^. In Fig. 2b, it is the local heat source term in the thermal diffusion equation which is revealed, and not temporally- and spatially broadened versions at time scales larger than the phonon relaxation time in the metal. This source term is of crucial importance for characterizing plasmonic devices, since it is weakly predictable with electromagnetic calculations without neglecting grain roughness or crevasse, which are crucial in absorption processes especially for highly confined SPPs^19^.

### Hot-spot absorption at the taper apex

**Fig. 3:**
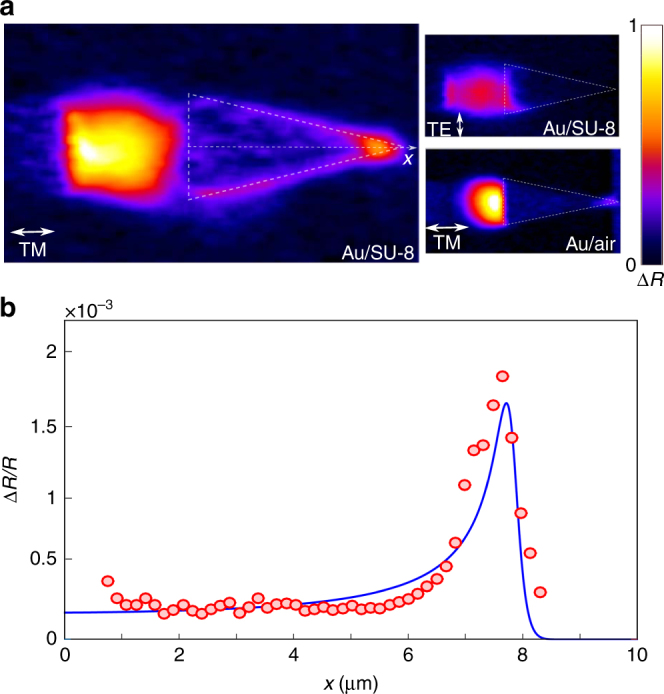
SPP damping rate enhancement at the tip apex. **a** Δ*R* image recorded for Δ*t* = 1 ps corresponding to a maximum signal at the taper apex. The superimposed thin-dashed lines show the taper contours. Uppermost inset: Δ*R* image recorded for a pump-beam polarized parallel to the slits. Lowermost inset: Δ*R* image recorded for TM polarization with a probe beam incident from air and focused on the air/Au interface. The Δ*R* images are 16 × 7 μm^2^ large and are displayed with the same color scale. **b** Lineshape of the normalized reflectance variation Δ*R*(*x*)/*R*(*x*) recorded along the taper longitudinal *x*-axis

To further analyze the hot spot at the apex, we focus our attention on thermoreflectance signals recorded at Δ*t* = 1 ps (Fig. 3a), just after the initial SPP decay via the creation of electron–hole pairs and before phonon relaxation takes place. Figure 3a additionally shows two companion records obtained for a 90° rotation of the pump-beam polarization (uppermost inset) and for a probe beam incident from air and focused at the air/Au surface (lowermost inset). Predictably, the signal is null for a TE-polarized pump with an electric field parallel to the slits. More interesting is the response obtained in the lowermost inset. No signal is visible at the lateral edges, whereas the hot spot, albeit weak, is again observed at the apex. This is intuitively understood by considering the spatial distribution of absorption. The SPP launched at the SU-8/Au interface is dominantly absorbed at the lateral edges, the SPP-mode profile broadens at the apex, and absorption takes place through the entire thickness of the gold film. This point will be discussed later.

Until now, we have displayed raw data of Δ*R*. However, the Δ*R* variations recorded at the apex are induced by permittivity changes dominantly occuring within a localized area (≈30 × 30 nm^2^), significantly smaller than the probe waist (≈400 × 400 nm^2^). Quite the contrary, the Δ*R* variations away from the taper edges are due to permittivity changes that cover the entire probe beam. In other words, since the effective taper surface intersected by the probe beam reduces as the probe is scanned toward the apex, raw ∆*R* data cannot faithfully represent the actual hot-carrier generation enhancement at the apex. This leads us to consider relative reflectivity variation signals ∆*R*/*R* for a fairer representation.

The red dots in Fig. 3b show ∆*R*/*R* data recorded as the probe beam is scanned along the taper longitudinal *x*-axis of symmetry. Consistently with previous results, which reported a large plasmon-to-hot-electron conversion efficiency in a conical nanofocusing gold structure^14^, a large ≈10-fold enhancement of the thermoreflectance signal is observed at the apex.

The full-width at half-maximum of the measured hot spot (≈800 nm) corresponds to the size of the probe beam, rather than the actual SPP decay hot spot size at the apex. Clearly, the finite spatial resolution of the probe prevents us from observing the actual hot-carrier generation enhancement at the apex. By assuming that 50% of the SPP absorption occurs along the lateral edges of the taper (as deduced from the thermoreflectance data) and that the tip curvature radius is 25 nm, simple geometrical considerations based on a spatial overlap between the tip and the Gaussian probe beam lead us to estimate a more realistic value of 30 for the local absorption enhancement, which is about three times larger than the measured one. The SPP-field enhancement at the taper apex is well known and understood as resulting from a progressive slowdown of the SPP in the last 10s of nanometers of the apex^16^. The added significance of the recorded thermoreflectance images is to directly evidence that the slowdown is accompanied by an SPP absorption enhancement.

### Hot-carrier relaxation dynamics

**Fig. 4:**
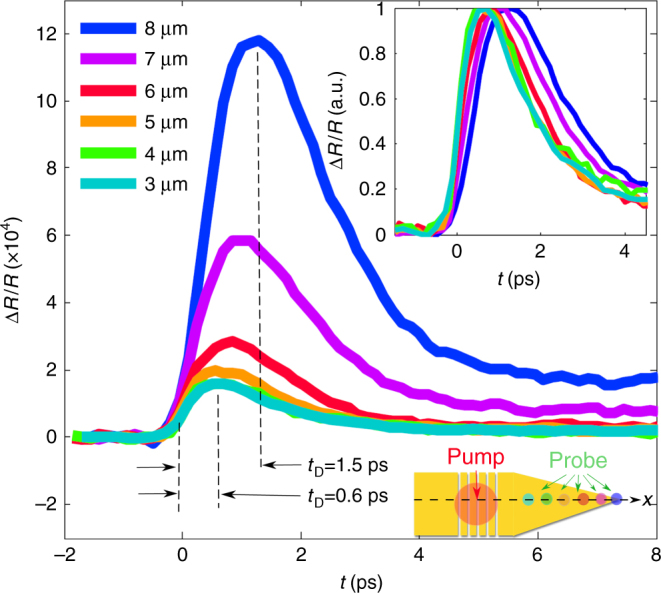
Time-resolved hot-carrier dynamics. Position-dependent transient Δ*R*/*R* signals obtained for six positions of the probe beam, focused at *x* = 3, 4 … 8 µm on the SU-8/Au interface by scanning the pump–probe delay. For *x* = 8 µm, the probe is focused on the apex, as indicated by the lowermost inset. The uppermost inset shows normalized Δ*R*/*R* signals

Let us now consider the hot-carrier relaxation during SPP focusing. Figure 4 shows the time-resolved responses recorded at the SU-8/Au interface for six focusing positions, *x* = 3, 4, … 8 µm, of the probe beam along the taper *x*-axis. The responses share the same general behavior^27,29^. First, Δ*R*/*R* rises up, due to intraband excitation of conduction electrons by absorption of SPPs of 1.55 eV (*λ*_0_ = 800 nm). The hot-carrier distribution, initially highly nonthermal, relaxes and part of the energy is absorbed by the free electron gas through electron–electron collisions, with a typical mean-free time between collisions for each electron ranging from 10 to 100 fs depending on the energy of the electron relative to the Fermi level. Scattering with phonons in the noble metals also occurs with a mean-free time from 10 to 30 fs, but the energy transferred in each collision is limited by the small phonon Debye energies, so that the net energy transfer to the lattice occurs at much smaller time scales at ~1 ps. Finally, a classical heat diffusion transport takes place with a much longer characteristic time.

The responses result from a rich variety of classical and quantum effects that govern the SPP hot-carrier generation and relaxation^27,30^. Interestingly, we note that the rising time *t*_D_ of Δ*R*/*R* varies with the position of the probe. For probes focused away from the apex, *x* = 3–5 µm, *t*_D_ is approximately equal to 500 fs, consistently with previous observations^17^, and recent ones are made with the same setup for gold thin films fabricated with the same process and with the same grating without a taper^19^. As the probe is focused at the apex, for *x* = 8 µm, the rising time is markedly larger, *t*_D_ ≈ 1.5 ps, suggesting that confinement causes the generation of a much stronger and longer-lived hot-electron bath, as confirmed by computational results hereafter.

Intuitively, the increase in rising time may have two main origins. The first one is a strong increase of the effective electron temperature at the apex, which causes a change of the electron and phonon thermal properties and of the electron–phonon coupling constant^31^. Several recent observations and predictions^32,33^ seem to promote drastic modifications of the ultrafast temporal responses of electron gas induced by changes in the energetic distribution of hot carriers generated in hot spots^20,34–37^. The second source is related to an increase of the SPP confinement at the apex, which modifies the initial spatial distribution of hot carriers before any electron–electron or electron–phonon relaxation processes take place, giving rise to carrier diffusion processes close to the apex.

### Two-temperature model

To better understand and distinguish the respective impact of the energetic and spatial distributions of the SPP-generated hot carriers on the anomalous rising time increase, we adopt, like in conventional analyses of thermoreflectance measurements, the two-temperature model (TTM). By assuming a local thermal equilibrium, the TTM tracks the dynamics of the temperatures of the electronic and lattice systems, *T*_e_ and *T*_1_, with a simple system of coupled nonlinear diffusion equations^29^

2a $$\frac{{\partial N}}{{\partial t}} = \frac{{ - N}}{{\tau _{{\mathrm{el}}}}} - \frac{N}{{\tau _{{\mathrm{el}} - {\mathrm{ph}}}}} + P_{\mathrm{w}}\left( {x,y,t} \right),$$

2b $$C_{\mathrm{e}}\left( {T_{\mathrm{e}}} \right)\frac{{\partial T_{\mathrm{e}}}}{{\partial t}} = \frac{\partial }{{\partial x}}\left( {K_{xx,{\mathrm{e}}}\frac{\partial }{{\partial {\mathrm{x}}}}T_{\mathrm{e}}} \right) + \frac{\partial }{{\partial y}}\left( {K_{yy,{\mathrm{e}}}\frac{\partial }{{\partial y}}T_{\mathrm{e}}} \right) \\ - g(T_{\mathrm{e}})\left( {T_{\mathrm{e}} - T_{\mathrm{l}}} \right) + \frac{N}{{\tau _{{\mathrm{el}}}}},$$

2c $$C_{\mathrm{l}}\frac{{\partial T_{\mathrm{l}}}}{{\partial t}} = \frac{\partial }{{\partial x}}\left( {K_{xx,{\mathrm{l}}}\frac{\partial }{{\partial x}}T_{\mathrm{l}}} \right) + \frac{\partial }{{\partial y}}\left( {K_{yy,{\mathrm{l}}}\frac{\partial }{{\partial y}}T_{\mathrm{l}}} \right) \\ + g\left( {T_{\mathrm{e}}} \right)\left( {T_{\mathrm{e}} - T_{\mathrm{l}}} \right) + \frac{N}{{\tau _{{\mathrm{el}} - {\mathrm{ph}}}}}.$$

Instead of a brute-force numeric approach coupling the model to time-dependent Maxwell equation solutions in 3D, we adopt a simpler 2D approach neglecting the gradients in the short vertical direction and highlighting confinement and power-flow effects in the plane of the antenna. Briefly, Eq. 2a–c describe the transfer of absorbed SPP power density *P*_w_(*x*, *y*, *t*), to a nonthermal hot-electron energy *N*, which subsequently thermalizes with the electrons and the lattice, thereby changing *T*_e_ and *T*_1_; all these quantities vary with *x*, *y*, and *t*. We describe the various terms in detail below.

**Fig. 5:**
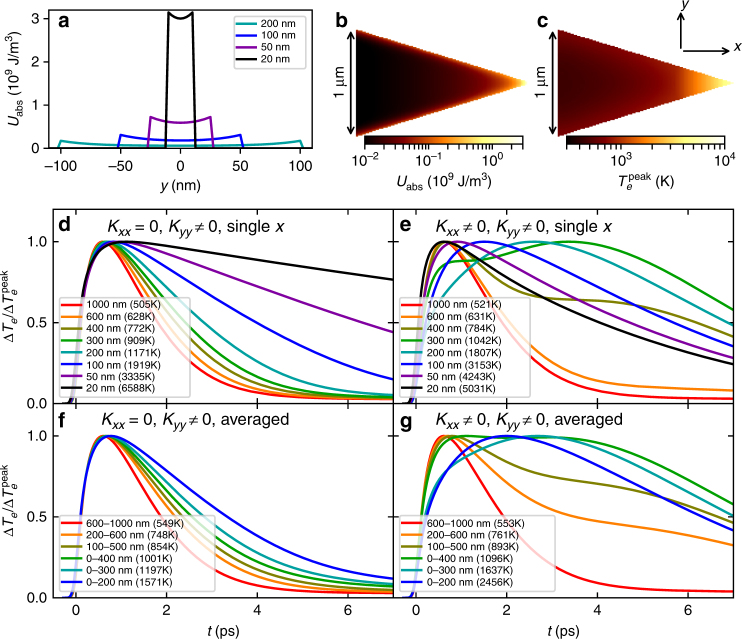
Impact of spatial confinement and heat flow on the rising time. Computed absorbed energy-density profile for an SPP at *λ*_0_ = 800 nm assuming 1-nJ pump pulse energy (**a**) for four taper widths and **b** over an area of the taper close to the apex. **c** shows the corresponding peak electron temperature predicted by TTM simulations. **d**, **e** show the simulated temperature profiles at various widths accounting only for heat flow along *y* in **d** and fully accounting for both directions in **e**. **f**, **g** show the corresponding temperatures averaged over ranges of widths, to account for the finite size of the probe beam. To show the rise and fall times clearly, the temperature profiles in **d**–**g** are normalized to their peak temperatures, which are indicated in parenthesis in the legends. Comparing **g**–**f** shows that heat flow from the apex with enhanced absorption due to SPP slowdown is responsible for the observed increase in rising time

First, we compute the SPP modes of the taper with an accurate Maxwell’s mode solver^38^. For every width, we normalize the modes so that their power flow is one. Since the *z*-dependence of the absorption-distribution profiles weakly depends on the taper width, we further average the absorbed power density $\frac{{ - \omega }}{2}{\mathrm{Im}}\left( {\varepsilon _{{\mathrm{Au}}}} \right)E_{{\mathrm{SP}}}^2$ along the vertical *z*-direction. We get *P*_w_(*x*, *y*, *t*) by multiplying this density at each time *t* by the instantaneous power density coupled into the SPP from the pump pulse, assuming the same illumination conditions as in the experiment (1-nJ Gaussian pulse, 150-fs FWHM). Figure 5a displays the corresponding absorbed energy density, $U_{{\mathrm{abs}}}(x,y) = {\int} {{\mathrm{d}}t\,P_{\mathrm{w}}(x,y,t)}$ for four widths, while Fig. 5b shows the same over the entire area. As expected, the confinement impacts both the energy-density magnitude and shape, starting from nonuniform profiles localized near the edges for large *w*’s to nearly uniform profiles with substantially higher energy densities for *w* < 50 nm.

Then, Eq. 2a describes the evolution of the nonthermal energy density *N*, which is fed by the absorbed power density. Electron–electron scattering internally thermalizes the electrons, transferring energy from *N* to thermal electrons at temperature *T*_e_ with time constant *τ*_el_. Similarly, electron–phonon scattering transfers energy directly from *N* to the lattices at temperature *T*_1_ with time constant *τ*_el−ph_. We assume typical values *τ*_el_ = 0.5 ps and *τ*_el−ph_ = 1 ps for these time constants^27^. Equation 2b and c describe the evolution of the electron and lattice temperatures, respectively, due to thermal conductivities *K*_e_ and *K*_1_, and electron–phonon coupling *g*. In reality, the *x* and *y* conductivities are equal, $K_{xx,{\mathrm{e}}} = K_{yy,{\mathrm{e}}} = K_{\mathrm{e}}$ (and similarly for *K*_1_), but below, we consider the effect of turning off one of the directions to isolate the cause of the rising-time enhancement at the apex. We use temperature-dependent electronic heat capacities *C*_e_(*T*_e_), electron–phonon coupling *g*(*T*_e_), and electronic thermal conductivities $K_{\mathrm{e}}\left( {T_{\mathrm{e}}} \right) = C_{\mathrm{e}}\left( {T_{\mathrm{e}}} \right)v_{\mathrm{F}}^2\tau _{\mathrm{e}}\left( {T_{\mathrm{e}}} \right){\mathrm{/}}3$ (*ν*_F_ is the Fermi velocity) derived from ab initio calculations^32^, which fully account for the band structure and the density of states and have recently been shown to provide quantitative agreement with spectral and temporal features in transient-absorption measurements^33^. We assume constant values based on an experiment for the lattice thermal conductivity *K*_1_ = 20 W/(mK) and heat capacity $C_{\mathrm{l}} = 2.4 \times 10^6$ J/(m^3^K), which play a much smaller role in the observed dynamics. We solve the coupled differential Eq. 2a–c using a finite-difference time-domain discretization scheme with 5-nm spatial resolution and an adaptive step-size embedded Runge–Kutta integrator for time evolution.

Figure 5d shows the simulated electron temperature profiles along the propagation *x*-axis for various widths, accounting only for heat conduction in the transverse *y*-direction, but not in the longitudinal *x*-direction by setting $K_{xx,{\mathrm{e}}} = K_{xx,{\mathrm{l}}} = 0$ in the TTM. Note the monotonic increase in the peak temperature with reducing width due to the increasing absorbed energy density. Correspondingly, the electron–phonon relaxation slows down due to the increased electron heat capacity at high temperatures, as is well known. This slowdown of the relaxation leads to a slightly longer rising time (by ~300 fs) for the narrowest widths.

Figure 5e shows the simulated temperature profiles accounting for conduction in both directions. The behavior is similar to the previous case for large widths, but fundamentally different for widths *w* ≤ 400 nm. The profiles first develop a shoulder at long times for *w* ~ 40 nm, which appears at earlier times with decreasing widths, until it becomes a single broader peak for 200-nm widths and below. This shoulder is caused by heat flow from the hotter apex toward the colder regions of the taper. Therefore, it appears at later times for larger widths (further from the apex). It also becomes weaker with increasing distance from the apex until it is no longer important beyond 400-nm widths. The result is a nonmonotonic increase followed by a decrease of the time to reach peak temperature, with reducing widths. This heat flow away from the apex also reduces the peak temperatures reached near the apex, thereby limiting the artificial increase in electron–phonon relaxation predicted in Fig. 5d with *y*-conductivity alone. Additionally, this heat flow makes the peak electron temperature shown in Fig. 5c more uniform spatially compared to the absorption profile in Fig. 5b.

The measurements do not show the nonmonotonicity in rising times discussed above because the width of the probe pulse results in averaging over the entire region containing the reverse heat flow. Figure 5f, g show the temperature profiles spatially averaged over ranges of widths, for the *y* only and both direction cases of conduction. Conduction in *y* alone shows the aforementioned small increase in rising time, while including the *x* conduction now exhibits the much larger increases in rising time, ~1 ps seen in an experiment.

The simple model of SPP absorption and electron thermalization already captures the key physics observed in our experiments: increased absorption at the apex due to SPP slowdown and heat flow toward regions of lower absorption over hundreds of nanometers and picosecond time scales. All TTM model parameters here are derived ab initio except the time scales *τ*_el_ and *τ*_el−ph_ of energy transfer from nonthermal electrons to thermal electrons and the lattice, respectively. In Supplementary Note 1, we show by solving the Boltzmann equation with ab initio collision integrals that these time scales do not increase with increasing absorbed energy density *U*_abs_, but rather decrease slightly. This may partly account for the TTM overestimating the increase in rising time due to power flow. Other effects not captured by the present model include further details of the electromagnetic field distributions, nonlocality of internal electron thermalization (electrons travel ~10–100 nm between the collisions that thermalize them), and modification of optical absorption due to the nonthermal electron distribution functions.

## Discussion

The understanding of how hot-carrier generation and relaxation are impacted by geometrical and physical effects, e.g., plasmon focusing, sharp apex, distribution function evolution, and electron–electron scattering processes, is critical. The electron dynamics and the associated lifetimes are important not just for SPP-induced hot-carrier technologies^1–4^, but for other modern prospects in plasmonics as well, e.g., ultrafast control of nanoantennas and molecules^39^, nonlinear generation of hot carriers, and nonlocal effects in plasmonic dimers^40^.

However, the time and length scales of carrier transport in metallic nanostructures are not well understood^28^ and have been the subject of recent debate^41,42^. Experimental studies of the electron–electron interaction dynamics in noble metal nanoparticles^43^ have shown that the electron collision rate increases with the hot-carrier density, which is in agreement with Fermi liquid theory, resulting in a faster internal electron thermalization. On the contrary, it has also been suggested^2^ that the lifetime of the initially generated nonthermal hot electrons can be substantially longer in nanostructures with respect to bulk because high nonthermal carrier density reduces electron–electron interactions^44–46^.

Because they are performed rigorously under the same experimental conditions with a device that offers a continuous transformation from an extended metal film to a nanoscale confinement at the apex, the present measurements unambiguously reveal any effect induced by confinement. They additionally confirm the extraordinary capability of adiabatic SPP tapers to effectively generate hot carriers^14^ at the apex and substantially modify the hot-carrier relaxation dynamics due to strong spatial inhomogeneity. The 1.5-ps rising time measured in the present work is much longer than the electron thermalization time and is also longer than those generally observed in small Au nanoparticles^15^. Additionally, adiabatic tapers may collect and funnel energy much more efficiently than isolated particles with the potential to boost quantum and nonequilibrium effects. In future work, carefully designed taper geometries could therefore generate even longer-lived hot carriers, essential for applications requiring carriers to survive long enough for charge transfer processes to occur^8,10,47,48^.

### Data availability

All data generated or analyzed during this study are available from the authors.

## Electronic supplementary material

Supplementary Information

Description of Additional Supplementary Files

Supplementary Movie 1

## References

[1] Atwater HA, Polman A (2010). Plasmonics for improved photovoltaic devices. Nat. Mater..

[2] Brongersma ML, Halas NJ, Nordlander P (2015). Plasmon-induced hot carrier science and technology. Nat. Nanotechnol..

[3] Chalabi H, Brongersma ML (2013). Plasmonics: harvest season for hot electrons. Nat. Nanotechnol..

[4] Clavero C (2014). Plasmon-induced hot-electron generation at nanoparticle/metaloxide interfaces for photovoltaic and photocatalytic devices. Nat. Photonics.

[5] Lal S, Clare SE, Halas N (2008). Nanoshell-enabled photothermal cancer therapy: impending clinical impact. J. Acc. Chem. Res..

[6] Huang X, Neretina S, El-Sayed MA (2009). Gold nanorods: from synthesis and properties to biological and biomedical applications. Adv. Mater..

[7] Awazu K (2008). A plasmonic photocatalyst consisting of silver nanoparticles embedded in titanium dioxide. J. Am. Chem. Soc..

[8] Mukherjee S (2012). Hot electrons do the impossible: plasmon-induced dissociation of H_2_ on Au. Nano. Lett..

[9] Linic S, Christopher P, Ingram DB (2011). Plasmonic-metal nanostructures for efficient conversion of solar to chemical energy. Nat. Mater..

[10] Knight MW, Sobhani H, Nordlander P, Halas NJ (2011). Photodetection with active optical antennas. Science.

[11] Garcıa de Arquer FP, Mihi A, Kufer D, Konstantatos G (2013). Photoelectric energy conversion of plasmon-generated hot carriers in metal-insulator-semiconductor structures. ACS Nano.

[12] Tisdale WA (2010). Hot-electron transfer from semiconductor nanocrystals. Science.

[13] Besteiro LV, Govorov AO (2016). Amplified generation of hot electrons and quantum surface effects in nanoparticle dimers with plasmonic hot spots. J. Phys. Chem. C.

[14] Giugni A (2013). Hot-electron nanoscopy using adiabatic compression of surface plasmons. Nat. Nanotechnol..

[15] Link S, El-Sayed MA (1999). Spectral properties and relaxation dynamics of surface plasmon electronic oscillations in gold and silver nanodots and nanorods. J. Phys. Chem. B.

[16] Stockman MI (2004). Nanofocusing of optical energy in tapered plasmonic waveguides. Phys. Rev. Lett..

[17] Van Exter M, Lagendijk A (1988). Ultra-short surface plasmon and phonon dynamics. Phys. Rev. Lett..

[18] Dilhaire S, Pernot G, Calbris G, Rampnoux JM, Grauby S (2011). Heterodyne picosecond thermoreflectance applied to nanoscale thermal metrology. J. Appl. Phys..

[19] Lozan O (2014). Anomalous light absorption around subwavelength apertures in metal films. Phys. Rev. Lett..

[20] Harutyunyan H (2015). Anomalous ultrafast dynamics of hot plasmonic electrons in nanostructures with hot spots. Nat. Nanotechnol..

[21] Verhagen E, Polman A, Kuipers L (2008). Nanofocusing in laterally tapered plasmonic waveguides. Opt. Express.

[22] Palik, E. D. *Handbook of Optical Constants of Solids* (Academic Press, New York, 1985).

[23] Lalanne P, Hugonin JP, Rodier JC (2005). Theory of surface plasmon generation at nanoslit aperture. Phys. Rev. Lett..

[24] Yang, J., Hugonin, J. P. & Lalanne, P. Near-to-far field transformations for radiative and guided waves. *ACS Photonics* **3**, 395–402 (2016).

[25] Lagarias JC, Reeds JA, Wright MH, Wright PE (1998). Convergence properties of the nelder-mead simplex method in low dimensions. SIAM J. Optim..

[26] Voisin C, Del Fatti N, Christofilos D, Vallée F (2001). Ultrafast electron dynamics and optical nonlinearities in metal nanoparticles. J. Phys. Chem. B.

[27] Sun CK, Vallée F, Acioli LH, Ippen EP, Fujimoto JG (1993). Femtosecond investigation of electron thermalization in gold. Phys. Rev. B.

[28] Desiatov B, Goykhman I, Levy U (2014). Direct temperature mapping of nanoscale plasmonic devices. Nano. Lett..

[29] Hohlfeld J (2000). Electron and lattice dynamics following optical excitation of metals. Chem. Phys..

[30] Groeneveld RH, Sprik R, Lagendijk A (1995). Femtosecond spectroscopy of electron-electron and electron-phonon energy relaxation in Ag and Au. Phys. Rev. B.

[31] Kittel C (2005). Introduction to Solid State Physics.

[32] Brown AM, Sundararaman R, Narang P, Goddard WA, Atwater HA (2016). Ab initio phonon coupling and optical response of hot electrons in plasmonic metals. Phys. Rev. B.

[33] Brown AM (2017). Experimental and *ab initio* ultrafast carrier dynamics in plasmonic nanoparticles. Phys. Rev. Lett..

[34] Manjavacas A, Liu JG, Kulkarni V, Nordlander P (2014). Plasmon-induced hot carriers in metallic nanoparticles. ACS Nano.

[35] Govorov AO, Zhang H (2015). Kinetic density functional theory for plasmonic nanostructures: breaking of the plasmon peak in the quantum regime and generation of hot electrons. J. Phys. Chem. C.

[36] Méjard R (2016). Energy-resolved hot-carrier relaxation dynamics in monocrystalline plasmonic nanoantennas. ACS Photonics.

[37] Demichel O (2016). Dynamics, efficiency, and energy distribution of nonlinear plasmon-assisted generation of hot carriers. ACS Photonics.

[38] Hugonin JP, Lalanne P, Del Villar I, Matias IR (2005). Fourier modal methods for modeling optical dielectric waveguides. Opt. Quant. Electron..

[39] Piatkowski L, Accanto N, van Hulst NF (2016). Ultrafast meets ultrasmall: controlling nanoantennas and molecules. ACS Photonics.

[40] Teperik TV, Nordlander P, Aizpurua J, Borisov AG (2013). Quantum effects and nonlocality in strongly coupled plasmonic nanowire dimers. Opt. Express.

[41] Khurgin JB, Boltasseva A (2012). Reflecting upon the losses in plasmonics and metamaterials. MRS Bull..

[42] Moskovits M (2015). The case for plasmon-derived hot carrier devices. Nat. Nanotechnol..

[43] Ertel K (1999). Time-resolved two-photon photoemission spectroscopy of HOPG and Ag nanoparticles on HOPG. Appl. Phys. B.

[44] Hartland GV (2011). Optical studies of dynamics in noble metal nanostructures. Chem. Rev..

[45] Sun CK, Vallée F, Acioli LH, Ippen EP, Fujimoto JG (1994). Femtosecond-tunable measurement of electron thermalization in gold. Phys. Rev. B.

[46] Groeneveld RH, Sprik R, Lagendijk A (1995). Femtosecond spectroscopy of electron-electron and electron-phonon energy relaxation in Ag and Au. Phys. Rev. B.

[47] Goykhman I, Desiatov B, Khurgin J, Shappir J, Levy U (2011). Locally oxidized silicon surface-plasmon Schottky detector for telecom regime. Nano. Lett..

[48] Scales C, Breukelaar I, Charbonneau R, Berini P (2011). Infrared performance of symmetric surface-plasmon waveguide Schottky detectors in Si. J. Lightwave Technol..

