# Supplementary material for: Increased rise time of electron temperature during adiabatic plasmon focusing
Source: Nat Commun. 2017 Nov 21;8:1656. doi: 10.1038/s41467-017-01802-y (PMC5698320; doi:10.1038/s41467-017-01802-y)
Supplement: Supplementary file 1 — Supplementary Information [file 41467_2017_1802_MOESM1_ESM.pdf]

Supplementary Note 1: Thermalization time of photo-excited electrons in gold

The TTM used to qualitatively interpret the experimental data assumes a thermal equilibrium for the electron bath. Here we use a much more advanced theoretical model to estimate the thermalization time of the electrons, i.e. the time required for an initial electronic distribution out of equilibrium to becomes a Fermi Dirac, in the experimental conditions used of the experiment. We show that the characteristic time constants for energy transfer from non-thermal electrons to thermalized electrons is much smaller than the measured rising time and reduces with increasing absorbed energy. This implies that the thermal equilibrium assumption of the TTM can be used with confidence, and is all the more accurate as the absorption occurs close to the apex in the experiment.

For that purpose, we apply the parameter-free *ab initio* methodology in [1] to simulate the photo-excitation and subsequent thermalization of electrons in gold. This method accounts for detailed band-structure effects in the electron and phonon density of states, carrier distributions as well as in the electron-phonon interaction matrix elements. The time evolution is obtained by solving the nonlinear time-dependent Boltzmann equation for spatially-independent energy distributions, assuming that the phonons are always approximately in equilibrium with each other at some lattice temperature T_l. The outputs of the simulation are the time-dependent electron occupation functions $f(E,t)$. We use a pump photon energy of 1.55 eV and a Gaussian 150-fs FWHM pulse , to match the experimental conditions.

Supplementary Fig. 1 shows the computed non-equilibrium distributions for an absorbed energy density $U_\mathrm{abs} = 5\times10^8$ J/m^3. Similar computations have been performed for U_abs varying from 10^7 to 10^9 J/m^3. The red curves represent the actual computed distributions, and the black curves are obtained by fitting Fermi-Dirac distributions to the computed non-equilibrium distributions, thus defining an effective electron temperature T_e.

We then calculate the energy density (N) of the non-thermal electrons as the difference between the electronic energy density of the non-equilibrium distribution and the fitted Fermi distribution. Finally, we take derivatives of the thermal electron and lattice energy densities to obtain the net rate of energy transfer into these subsystems. Using the values of the electron-phonon coupling constant g used in the TTM (see main text), we then infer the rate of energy transfers from non-thermal electrons to thermal electrons ($d(N \to e)/dt$) and the lattice ($d(N \to l)/dt$).

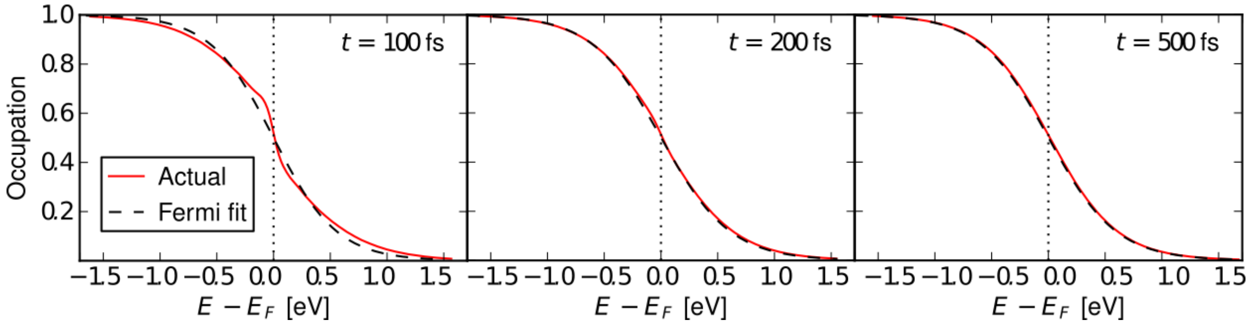

Supplementary Figure 1. Distribution function of free electrons in gold at three time delays after the pump pulse, $t = 100$, 200 and 500 fs. These theoretical results are obtained by solving the nonlinear time-dependent Boltzmann equation for spatially-independent energy distributions using *ab initio* collision integrals and excited electron distributions. Results are shown here for a Gaussian laser pulse with 150 fs FWHM, 1.55 eV photon energy (800-nm wavelength), and an absorbed energy density $U_\mathrm{abs} = 5\times10^8$ J/m^3.

Notice that the rate of energy transfers to e and l are not directly proportional to N, so the time constants assumed for the extended TTM have to correspond to an average value. The instantaneous rate, $\tau^{-1} = N^{-1}dN/dt$, can be further averaged weighted by N to get $\tau^{-1} = \int dN / \int N dt$, and finally the effective time constants for the relaxation of non-thermal electrons to thermal electrons ($T_{\mathrm{N}\to\mathrm{e}}$) and to the lattice ($T_{\mathrm{N}\to\mathrm{l}}$) are calculated. We report these effective time constants as a function of the absorbed energy density in Table 1. Note that $T_\mathrm{e}^\mathrm{max}$ is the electron temperature that would arise if all the absorbed energy were deposited into only the thermal electrons, while $T_\mathrm{e}^\mathrm{peak}$ is the peak electron temperature reached in the simulations (estimated via Fermi function fitting).

U_{abs} [J/m^3]	T_e^{max} [K]	T_e^{peak} [K]	τ_{tot} [ps]	$T_{N\to e}$ [ps]	$T_{N\to l}$ [ps]
1x10^7	638	371	1.012	2.001	2.049
2x10^7	852	459	1.000	1.834	2.197
5x10^7	1301	770	0.914	1.372	2.735
1x10^8	1823	1281	0.756	0.958	3.586
2x10^8	2572	2047	0.587	0.670	4.756
5x10^8	3985	3536	0.355	0.376	6.185
1x10^9	5335	5301	0.029	0.029	3.161

Supplementary Table 1. Non-equilibrium electron relaxation parameters extracted from *ab initio* Boltzmann simulations for several values of absorbed energy density in bulk gold. All parameters are defined in the text. τ_{tot} represents the overall internal thermalization time of the electrons in bulk gold, $1/\tau_{tot} = 1/T_{N\to e} + 1/T_{N\to l}$. These local / bulk values are well below the ps values observed in experiment; the long rise times observed must therefore be due to spatial transport / diffusion effects as discussed in the main text.

With increasing absorbed energy density, electron thermalization becomes more rapid because a larger fraction of electrons are already excited, and their first collisions more strongly affect the slope of the electron occupation function near the Fermi level (which dominates the extracted T_e, and also the optical signature due to $d \to s$ transitions). The relative importance of energy transfer directly from non-equilibrium electrons to the lattice correspondingly diminishes. This trend continues till an absorbed power of 10^9 J/m^3, beyond which the number of excited electrons is large enough to saturate the distribution function (all electrons in an energy range are excited), and separating the observed distributions into thermal and non-thermal components becomes ill-defined.

Supplementary Note 2: Impact of the pump power on the rise time

In the main text, we interpret the significant increase of t_D as arising from (i) a strong increase of the effective electron temperature at the apex, which causes a change of the electron and phonon thermal properties and of the electron-phonon coupling constant [2], and (ii) an increase of the SPP confinement at the apex, which modifies the initial *spatial* distribution of hot carriers before any electron-electron or electron-phonon relaxation processes take place, giving rise to carrier diffusion processes close to the apex. In this Section, we confirm the interpretation (i) with experimental data showing that the rise time increases with the pump power incident onto the grating coupler.

Supplementary Fig. 2 shows the thermoreflectance signals measured at the taper apex for several pump powers. We could not vary significantly the power, because very quickly we are damaging the grating region of the taper (our measurements are all performed for power close to the limit). A clear increase of the rise time is observed as the pump power is increased (from $P = 20$ to 80 µW before microscope objective), which qualitatively support our interpretation (i).

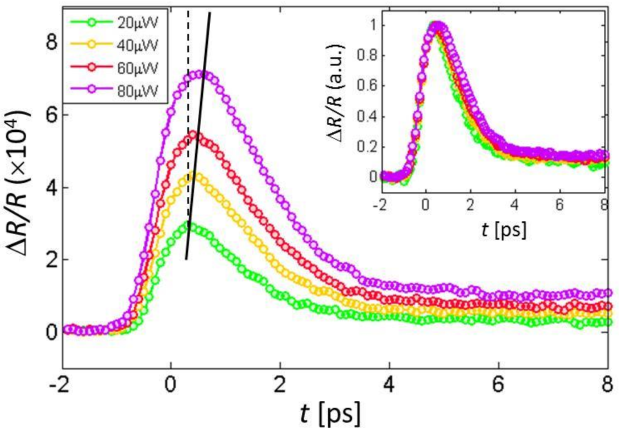

Supplementary Figure 2. Rising time dependence on the pump power. Transient *ΔR/R* signals obtained for 4 powers of the pump beam, focused at tip apex on the SU-8/Au interface by scanning the pump-probe delay. The solid black line locates the *ΔR/R* peaks and the dashed vertical line is plotted for convenience. The inset shows normalized *ΔR/R* signals. Note that the time origin is arbitrary.

Supplementary References

[1] Brown, A. M., Sundararaman, R., Narang, P., Schwartzberg, A. M., Goddard, W. A., Atwater, H. A., Experimental and *ab initio* ultrafast carrier dynamics in plasmonic nanoparticles. Phys. Rev. Lett. **118**, 087401 (2017).
[2] Kittle, C., *Introduction to Solid State Physics*, (J. Wiley and Sons eds., New York, 2005).
