# Supplementary material for: Increased rise time of electron temperature during adiabatic plasmon focusing
Source: Nat Commun. 2017 Nov 21;8:1656. doi: 10.1038/s41467-017-01802-y (PMC5698320; doi:10.1038/s41467-017-01802-y)
Supplement: Supplementary file 2 — Description of Additional Supplementary Files [file 41467_2017_1802_MOESM2_ESM.pdf]

Description of Additional Supplementary Files

File Name: Supplementary Movie
Description: Movie of ΔR-images recorded at the SU-8/Au interface with a 67-fs time step during the SPP nano-focusing. Snapshots of the movie are presented in Fig. 2.
